# Quercetin-associated cardioprotection in preclinical myocardial ischemia-reperfusion injury: a systematic review and meta-analysis

**DOI:** 10.3389/fphar.2026.1863814

**Published:** 2026-07-22

**Authors:** Zhao Shi, Zheng Liang, Ding Chen, Xingye Wang, Dongze Zhang, Ming Yao, Lihong Jiang

**Affiliations:** 1 Changchun University of Chinese Medicine, Changchun, China; 2 Department of Cardiology, the Affiliated Hospital to Changchun University of Chinese Medicine, Changchun, China

**Keywords:** cardioprotection, meta-analysis, myocardial ischemia-reperfusion injury, preclinical studies, quercetin

## Abstract

**Background and objective:**

Prompt reperfusion is the standard treatment for acute myocardial infarction, but it can induce myocardial ischemia-reperfusion injury (MIRI). Quercetin, a plant-derived flavonoid, has been investigated as a potential cardioprotective agent in preclinical MIRI models; however, its effect magnitude, dose-exposure relevance, and mechanistic specificity remain uncertain. This systematic review and meta-analysis evaluated quercetin monotherapy in vivo and *ex vivo* MIRI models.

**Methods:**

Databases were searched from inception to March 2026 for controlled preclinical studies comparing quercetin monotherapy with vehicle-treated MIRI controls. The primary endpoint was myocardial infarct size. Secondary outcomes included myocardial injury biomarkers, hemodynamic recovery, oxidative-stress markers, and inflammatory cytokines. Random-effects models calculated standardized mean differences (SMDs) or mean differences (MDs). Subgroup, leave-one-out, split-control, risk-of-bias-informed, dose-response meta-regression, and publication-bias analyses were performed where feasible. This review was registered in the International Prospective Register of Systematic Reviews (PROSPERO; CRD420261325366).

**Results:**

Twenty studies were included. Quercetin was associated with reduced infarct size compared with vehicle controls (SMD = −2.98, 95% confidence interval −4.37 to −1.58; P < 0.00001). A split-control sensitivity analysis showed an attenuated but directionally consistent effect (SMD = −2.22, 95% confidence interval −3.16 to −1.28). Exploratory dose-response meta-regression did not identify a clear linear association between systemic dose and infarct-size effect. Quercetin was associated with an 18.35-percentage-point improvement in left ventricular developed pressure (LVDP) recovery and favorable changes in selected maximal rates of pressure change (±dP/dtmax). It was also associated with reduced myocardial injury biomarkers, decreased malondialdehyde (MDA), increased superoxide dismutase (SOD) and glutathione (GSH), and lower tumor necrosis factor-alpha (TNF-α), interleukin-1 beta (IL-1β), and interleukin-6 (IL-6). Exploratory subgroup analyses suggested attenuated responsiveness in comorbid or metabolically altered models, but this finding was limited and confounded. Egger testing suggested possible small-study effects for infarct size.

**Conclusion:**

Quercetin is associated with a directionally consistent preclinical cardioprotective signal in rodent *in vivo* and *ex vivo* MIRI models. However, the magnitude, mechanistic specificity, and translational relevance remain uncertain because of heterogeneity, possible publication bias, unclear methodological quality, limited pharmacokinetic exposure data, and scarce sex-balanced, comorbid, and large-animal evidence.

**Systematic Review Registration:**

https://www.crd.york.ac.uk/PROSPERO/view/CRD420261325366, identifier CRD420261325366.

## 1 Introduction

Acute myocardial infarction (AMI) remains a leading cause of morbidity and mortality globally, primarily characterized by the loss of functional cardiomyocytes and deterioration of cardiac pump function (Heusch, 2020). Currently, the prompt restoration of blood flow via primary percutaneous coronary intervention (PPCI) is established as the primary therapeutic modality to salvage ischemic myocardium (Zuccarelli et al., 2024). Paradoxically, reperfusion itself exacerbates tissue damage—a phenomenon defined as MIRI, which accounts for up to 50% of the final infarct size and contributes to adverse ventricular remodeling (Sagris et al., 2024). The pathogenesis of MIRI is complex and involves interacting processes, including an abrupt increase in reactive oxygen species (ROS), calcium overload, sterile inflammatory signaling, NOD-like receptor family pyrin domain-containing 3 (NLRP3) inflammasome activation, and pro-inflammatory cytokine responses (Sagris et al., 2024; Han et al., 2026). Given the multifactorial nature of MIRI and the limited clinical translation of single-target cardioprotective strategies, there remains interest in agents that may influence multiple injury-related pathways (Li et al., 2022).

Recently, the cardiovascular and cardioprotective potential of plant-derived flavonoids, including quercetin, has attracted increasing attention (Kozłowska and Szostak-Węgierek, 2022). Quercetin (3,5,7,3′,4′-pentahydroxyflavone), a plant-derived flavonoid widely distributed in fruits and vegetables, has been reported to exert antioxidant, anti-inflammatory, and cytoprotective effects in experimental cardiovascular models (Zhang et al., 2023; Ozorowski et al., 2025). Preclinical studies and mechanistic reviews suggest that quercetin may modulate oxidative-stress responses, mitochondrial preservation, and inflammatory signaling pathways relevant to MIRI (Azizidoost et al., 2025). In addition, selected studies have linked quercetin to survival- and energy-sensing pathways, including sirtuin 1 (SIRT1) and adenosine monophosphate-activated protein kinase (AMPK) signaling, although these mechanisms require context-specific validation in MIRI models (Chen J. et al., 2025).

A critical appraisal of the available preclinical evidence for quercetin is therefore needed. A previous meta-analysis (Siti et al., 2022) provided preliminary insights; however, it was constrained by methodological limitations. Notably, it omitted the assessment of myocardial infarct size—a key morphological endpoint in preclinical cardioprotection studies. Furthermore, it aggregated disparate hemodynamic metrics with varying physiological implications and measurement units into a single analysis, thereby complicating physiological interpretation and contributing to statistical heterogeneity. Although a single diabetic study was included, targeted subgroup analyses were not performed, leaving uncertainty regarding whether baseline metabolic comorbidities may modify quercetin responsiveness in experimental MIRI models (Ferdinandy et al., 2023). Emerging evidence suggests that these pre-existing pathological conditions may alter myocardial redox homeostasis and blunt the efficacy of cardioprotective interventions (Chen H. et al., 2025; Mustafa et al., 2025).

To address these gaps, we conducted a systematic review and meta-analysis of quercetin-associated cardioprotection in preclinical MIRI models. We evaluated myocardial infarct size as the primary endpoint and assessed myocardial injury biomarkers, hemodynamic recovery, oxidative-stress markers, and inflammatory outcomes as supportive secondary endpoints. Through pre-specified subgroup analyses, we explored whether experimental model type and baseline metabolic comorbidities were associated with differences in quercetin responsiveness. These findings may inform future preclinical study design and translational evaluation.

## 2 Methods

### 2.1 Study design and registration

This systematic review and meta-analysis was prospectively registered with the International Prospective Register of Systematic Reviews (PROSPERO; registration number: CRD420261325366) in accordance with the Navigating PROSPERO4animals guidelines (Bannach-Brown et al., 2024). The study design, conduct, and reporting followed the Preferred Reporting Items for Systematic Reviews and Meta-Analyses (PRISMA) 2020 statement (Page et al., 2021). Since this review evaluates quercetin as a plant-derived flavonoid intervention rather than a botanical drug preparation, botanical authentication was not applicable. Nevertheless, relevant elements of the Four Pillars principles were addressed by focusing on *in vivo* and *ex vivo* pharmacological models, vehicle-controlled comparisons, explicit reporting of dose and route of administration, and cautious interpretation of translational relevance (Heinrich et al., 2022).

### 2.2 Search strategy

This systematic review and meta-analysis was conducted in accordance with the PRISMA guidelines. A comprehensive literature search was performed across databases including PubMed, Web of Science, Embase, Cochrane Library, China National Knowledge Infrastructure (CNKI), Chinese Science and Technology Journal Database (VIP), WanFang Database, and China Biology Medicine (CBM) Database, from inception to March 2026. The inclusion criteria were defined as follows: (1) preclinical controlled studies utilizing *in vivo* or *ex vivo* (Langendorff) models of MIRI; (2) quercetin monotherapy in the experimental group; (3) presence of a vehicle-treated MIRI control group; and (4) reporting of at least one of the predefined primary or secondary quantitative outcomes. The exclusion criteria were defined as follows: (1) *In vitro* cell culture models, clinical trials, or *in silico* studies; (2) Non-reperfusion models or non-ischemic heart failure models; (3) Co-administration of quercetin with other active pharmacological agents or interventions; (4) Duplicate publications or studies with overlapping datasets; (5) Studies lacking extractable quantitative data that could not be resolved; (6) Non-original research including reviews, editorials, case reports, and conference abstracts.

### 2.3 Data extraction and quality assessment

Data extraction was performed independently by two reviewers. For outcomes presented exclusively in graphical format, numerical values were objectively extracted using GetData Graph Digitizer software. Reviewers were not blinded to study authors, institutions, or journals during screening and data extraction. Chinese-language full texts were screened and extracted by two reviewers fluent in Chinese, and extracted data were independently cross-checked against the original full texts.

To ensure methodological rigor and adhere to Cochrane guidelines, specific data extraction rules were pre-defined:

1. Handling of multiple dose groups: In studies where multiple quercetin dose or concentration groups were compared with a single shared control group, one comparison was selected for the primary analysis according to predefined extraction rules to avoid double-counting the shared control group. When the original study specified a principal pharmacological comparison, that comparison was used; otherwise, the highest systemic dose or the prespecified *ex vivo* concentration was selected. To assess the potential influence of this decision on the primary endpoint, an additional split-control sensitivity analysis was performed for myocardial infarct size. In this sensitivity analysis, all eligible quercetin monotherapy dose arms were retained as separate comparisons, and the sample size of the shared control group was divided across the corresponding comparisons.
2. Data transformation: In studies reporting data as mean ± standard error of the mean (SEM), values were converted to standard deviations (SDs) using the formula $\text{SD}=\text{SEM}\times \sqrt{n}$ .

The methodological quality and risk of bias of the included animal studies were assessed using the Systematic Review Centre for Laboratory animal Experimentation (SYRCLE) risk of bias tool.

### 2.4 Outcome measures

The primary outcome was myocardial infarct size. Myocardial injury biomarkers, including cardiac troponin I (cTnI), creatine kinase-MB (CK-MB), lactate dehydrogenase (LDH), and total creatine kinase (CK), were analyzed using outcome-specific univariate meta-analyses and were not combined into a single composite endpoint. Cardiac hemodynamic recovery was assessed via left ventricular developed pressure (LVDP) and the maximal rates of pressure change (±dP/dtmax). We extracted data on parameters of oxidative stress, including malondialdehyde (MDA), superoxide dismutase (SOD), and glutathione (GSH), as well as key mediators of the pro-inflammatory cytokine cascade, such as tumor necrosis factor-alpha (TNF-α), interleukin-1 beta (IL-1β), and interleukin-6 (IL-6). These predefined secondary outcomes are interpreted as supportive and mechanistic outcomes rather than multiple independent confirmatory efficacy endpoints.

### 2.5 Statistical analysis

All statistical analyses were performed using Review Manager (RevMan, version 5.4) and Stata software. Given the inherent variations in experimental designs, a random-effects model was selected for all pooled analyses.

1. Mean difference (MD) with 95% confidence intervals (CIs) was used for LVDP recovery rate. For + dP/dtmax and −dP/dtmax, absolute values reported in mmHg/s and baseline-adjusted recovery rates reported as percentages were analyzed as separate outcomes. Standardized mean difference (SMD) with 95% CIs was used for other continuous outcomes or when measurement scales varied across studies.
2. Subgroup analyses: Predefined outcome-specific subgroup analyses were performed to explore potential sources of inter-study heterogeneity. For myocardial infarct size: stratifications were performed based on pathological status (healthy vs. comorbidity models), experimental models (*in vivo* vs. *ex vivo*), and dosage regimens (low/medium doses, high doses, and *ex vivo* perfusion) to delineate the boundaries of cardioprotective efficacy. For CK-MB, analyses were stratified by experimental setting. An additional *post hoc* subgroup analysis by intervention timing was performed for infarct size only, using the same 11 comparisons included in the primary analysis. Timing categories were defined as prevention/preconditioning before ischemia/reperfusion (I/R), reperfusion-onset/postconditioning treatment, and post-MIRI long-term treatment.
3. Additional study-level analyses: For infarct size, systemic quercetin dose was log10-transformed and analyzed as a study-level covariate in univariable random-effects meta-regression. *Ex vivo* Langendorff perfusate concentrations were not converted into mg/kg and were excluded from the continuous systemic-dose regression. Control-group mean infarct size was used as a study-level covariate in univariable random-effects meta-regression. Scatter plots were generated to visualize the relationships between control-group infarct size and both the SMD and the absolute infarct-size reduction, calculated as control mean minus quercetin mean. Sensitivity analyses excluding Challa et al., 2011 were conducted for both exploratory meta-regression analyses.
4. Composite display: For the pro-inflammatory cascade (TNF-α, IL-1β, IL-6), data were visually compiled into a composite forest plot with distinct subgroups for each cytokine. To maintain biological interpretability and prevent inappropriate statistical pooling of distinct molecular entities, the overall pooled effect size across different cytokines was deliberately omitted.

Heterogeneity was assessed using I^2^ and the Chi-square test. Leave-one-out sensitivity analyses were performed by sequentially omitting individual studies. To assess whether the handling of multi-dose studies influenced the primary endpoint, an additional split-control sensitivity analysis was performed for myocardial infarct size. For multi-arm studies in which multiple eligible quercetin monotherapy dose groups shared a common control group, each eligible quercetin dose group was retained as a separate comparison, while the sample size of the shared control group was divided across the corresponding comparisons. Control-group means and SDs were left unchanged. When integer sample sizes were required, the shared control group was divided as evenly as possible while preserving the original total control-group sample size. Risk-of-bias-informed sensitivity analyses were performed by excluding studies with explicit high-risk judgments in at least one SYRCLE domain when at least three studies remained for pooling. Potential publication bias was evaluated by funnel plot inspection and Egger’s regression test. For myocardial infarct size, Duval and Tweedie’s trim-and-fill analysis was performed as an exploratory sensitivity analysis of funnel-plot asymmetry. Statistical significance was set at P < 0.05.

## 3 Results

### 3.1 Literature search

**FIGURE 1:**
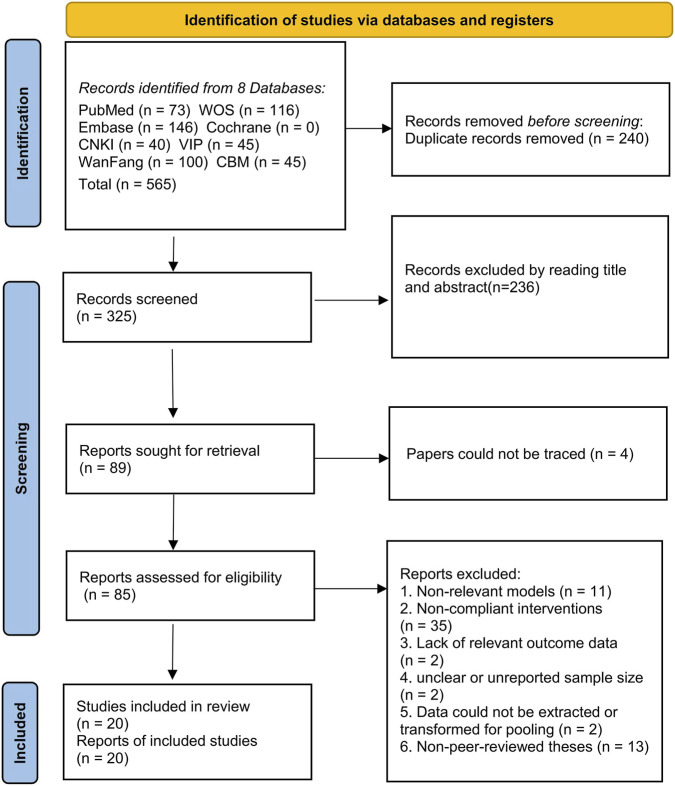
PRISMA 2020 flowchart for literature screening.

A total of 565 publications were initially retrieved through comprehensive electronic database searching. After removing 240 duplicate records, 325 records underwent title and abstract screening, which led to the exclusion of 236 irrelevant records. Subsequently, full texts were sought for the remaining 89 reports; however, four reports could not be retrieved. Therefore, full-text eligibility assessment was conducted for 85 articles. Based on the pre-specified inclusion and exclusion criteria, 65 articles were excluded for the following reasons: non-compliant interventions (n = 35), non-peer-reviewed theses (n = 13), non-relevant models (n = 11), lack of relevant outcome data (n = 2), unclear or unreported sample size (n = 2), and data that could not be extracted or transformed for pooling (n = 2). Finally, 20 independent preclinical studies (Brookes et al., 2002; Cao et al., 2004; Ikizler et al., 2007; Ahmed et al., 2009; Bartekova et al., 2010; Challa et al., 2011; Gan et al., 2011; Jin et al., 2012; Wang X. et al., 2013; Wang Y. et al., 2013; Zhang and Qian, 2015; Bartekova et al., 2016; Ferenczyova et al., 2020; Liu et al., 2020; He et al., 2021; Liu et al., 2021; Xuan et al., 2022; Tao et al., 2023; Domingos et al., 2025; Kandpal et al., 2025) met all criteria and were included in the systematic review and quantitative meta-analysis. The detailed selection process is illustrated in the PRISMA flow diagram (Figure 1).

### 3.2 Description of included studies

Sprague-Dawley rats were used in 11 studies, Wistar rats in eight studies, and Zucker Diabetic Fatty (ZDF) rats in one study. In terms of sex, both male and female animals were used in seven studies; one study used only female animals, and all other 12 studies used male animals. Eleven of the included studies used *in vivo* ligation-reperfusion modeling, and the remaining nine studies used global ischemia-reperfusion in isolated Langendorff-perfused hearts. The routes of administration varied, primarily including intragastric/gavage (n = 7), intravenous (n = 4), intraperitoneal (n = 4), and via Langendorff perfusate (n = 4), with per os/oral (n = 1) and drinking water (n = 1) also utilized. The systemic dosage ranged from 0.03 to 100 mg/kg, while the concentration in perfusate ranged from 0.1 to 15 μmol/L. In addition, the timing of quercetin administration varied, with treatments given prophylactically before ischemia/ligation, immediately before or at the onset of reperfusion, or chronically post-surgery. The duration of treatment ranged from a single acute administration up to 6 consecutive weeks. Across the included studies, comparators were primarily vehicle-treated MIRI controls; therefore, this review evaluates quercetin-associated effects relative to vehicle controls rather than comparative efficacy against an established MIRI-specific pharmacological positive-control agent. Table 1 presents the characteristics of the included studies.

**TABLE 1:** Characteristics of included studies.

Number	Study ID	Species (sex, n = experimental/control group)	Weight	Model (method)	Anesthetic	Treatment group (dosage)	Administration route	Control group	Outcomes
1	Jin et al. (2012)	Sprague–Dawley rats (male, n = 9/9)	200–220 g	Ligation for 30min, then reperfusion for 12 h	Ketamine (100 mg/kg, i.p.) and xylazine (10 mg/kg, i.p.)	Que (1.0 mg/kg), i.v	Injected 5 min before to ligation	Distilled water	1) Infarct size; 2) LVEDP; 3) +dP/dtmax; 4) -dP/dtmax; 5) TNF-α; 6) IL-10; 7) TNF-α mRNA and protein; 8) IL-10 mRNA and protein
2	Ferenczyova et al. (2020)	ZDF rats (male, n = 12/12)	487 ± 10 g	30-min global ischemia/120-min reperfusion in langendorff-perfused hearts	Thiopental (50 mg/kg, i.p.)	Que (20 mg/kg), p.o	Once a day for 6 consecutive weeks	Vehicle	1) SBP; 2) vascular function; 3) LVDP; 4) +dP/dtmax; 5) -dP/dtmax; 6) CF; 7) infarct size; 8) RISK pathway proteins; 9) FBG; 10) lipid profile
3	Wang Y. et al. (2013)	Sprague–Dawley rats (male, n = 20/20)	250 ± 20 g	Ligation for 30min, then reperfusion for 2 h	Pentobarbital sodium (40 mg/kg, i.p.)	Que (10 mg/kg), i.p	Injection 5 min before reperfusion	no treatment	1) Infarct size; 2) CK; 3) LDH; 4) TUNEL; 5) cleaved caspase-3; 6) protein expression (p-Akt, Akt, Bcl-2, Bax)
4	Domingos et al. (2025)	Wistar rats (female, n = 9/9)	285–336 g	30-min global ischemia/60-min reperfusion in langendorff-perfused hearts	Not mentioned	Que (25 mg/kg),i.g	Starting 7 days post-surgery, five times a week for 4 consecutive weeks	Vehicle	1) LVDP; 2) LVEDP; 3) +dP/dtmax; 4) -dP/dtmax; 5) infarct size
5	Zhang and Qian (2015)	Sprague–Dawley rats (male and female, n = 10/10)	180–220 g	Ligation for 30min, then reperfusion for 2 h	Pentobarbital sodium (i.p.)	Quercetin (25, 50, and 100 mg/kg), i.g	Starting 7 days pre-surgery, once a day for 7 consecutive days	Vehicle	1) Infarct size; 2) CK; 3) LDH; 4) TNF-α; 5) IL-1β
6	Wang X. et al. (2013)	Sprague–Dawley rats (male, n = 10/10)	300–350 g	Ligation for 30min, then reperfusion for 1 h	10% chloral hydrate (4 mL/kg, i.p.)	Quercetin (15, 30, and 60 mg/kg), i.g	Once a day for 1 consecutive week pre-surgery	Normal saline	1) Infarct size; 2) CK-MB; 3) cTnI; 4) TNF-α; 5) IL-6; 6) MDA; 7) SOD; 8) GSH-Px
7	Cao et al. (2004)	Wistar rats (male, n = 5/5)	250–300 g	Ligation for 10min, then reperfusion for 15min	20% urethane (1 g/kg, i.p.)	Quercetin (0.2, 1.0, and 5.0 mg/kg),i.v	Injected 5 min before to ligation	not mentioned	1) CPK; 2) GSH-px
8	Xuan et al. (2022)	Sprague–Dawley rats (male and female, n = 12/12)	250 ± 50 g	Ligation for 30min, then reperfusion for 2 h	2% pentobarbital sodium (i.p.)	Quercetin (25 and 50 mg/kg), i.v	Once a day for 4 consecutive weeks after surgery	Normal saline	1) Infarct size; 2) TUNEL; 3) ROS; 4) MDA; 5) SOD; 6) AMPK/TSC2/mTOR pathway protein expression
9	Liu et al. (2020)	Sprague–Dawley rats (male, n = 10/10)	220–250 g	Ligation for 30min, then reperfusion for 1 h	7% chloral hydrate (5 mL/kg, i.p.)	Quercetin (10 mg/kg), i.v	Injection 15 min before reperfusion	no treatment	1) IL-6
10	Tao et al. (2023)	Sprague–Dawley rats (male, n = 15/15)	250–300 g	Ligation for 30min, then reperfusion for 3 h	2.5% pentobarbital sodium (i.p.)	Quercetin (50 mg/kg), i.p	1 injection before reperfusion	Normal saline	1) Infarct size; 2) CK-MB; 3) cTnI; 4) TNF-α; 5) IL-6; 6) TUNEL
11	He et al. (2021)	Sprague–Dawley rats (male, n = 17/17)	180–250 g	Ligation for 30min, then reperfusion for 40min	10% urethane (i.p.)	Quercetin (25, 50, and 100 mg/kg),i.g	Once a day for 1 consecutive week pre-surgery	Normal saline	1) Infarct size; 2) LDH; 3) CK-MB; 4) IL-1β; 5) IL-6; 6) TNF-α; 7) MDA; 8) SOD
12	Ahmed et al. (2009)	Wistar rats (male, n = 10/10)	180–220 g	Ligation for 35min, then reperfusion for 10min	Urethane (1.4 g/kg, i.p.)	Quercetin (5 mg/kg), i.g	Once a day for 7 consecutive days before ligation	Vehicle	1) CK; 2) GSH
13	Challa et al. (2011)	Wistar rats (male and female, n = 6/6)	200–250 g	Ligation for 30min, then reperfusion for 4 h	Thiopental (30 mg/kg, i.p.)	Quercetin (5 and 10 mg/kg), i.p	Injection 10 min before reperfusion	Vehicle	1) Infarct size
14	Brookes et al. (2002)	Sprague–Dawley rats (male, n = 5/6)	∼250 g	Langendorff perfusion of isolated hearts with 22-min global ischemia and 30-min reperfusion	Pentobarbital sodium (50 mg/kg i.p.)	Quercetin (0.03 mg/kg),i.g	Once a day for 4 consecutive days	Normal saline	1) LVDP
15	Ikizler et al. (2007)	Sprague–Dawley rats (male, n = 10/10)	250–300 g	Langendorff perfusion of isolated hearts with 60-min global ischemia and 60-min reperfusion	Pentobarbital sodium (50 mg/kg i.p.)	Quercetin ((50 mg/kg,i.g. Chronic treatment) and (15 μmol/L in perfusate. Acute treatment))	Once a day for 7 consecutive days	Vehicle	1) +dP/dtmax; 2) CF; 3) LDH; 4) CK-MB; 5) cTnI; 6) MDA; 7) GSH
16	Bartekova et al. (2016)	Wistar rats (male and female, n = 6/6)	80–100 g	Langendorff perfusion of isolated hearts with 25-min global ischemia and 40-min reperfusion	Thiopental (50 mg/kg, i.p.)	Quercetin (20 mg/kg via drinking water)	Once a day for 4 consecutive weeks	Vehicle	1) LVDP; 2) +dP/dtmax; 3) -dP/dtmax; 4) CF
17	Liu et al. (2021)	Wistar rats (male and female, n = 7/7)	250 ± 20 g	Langendorff perfusion of isolated hearts with 30-min global ischemia and 55-min reperfusion	Pentobarbital sodium (60 mg/kg i.p.)	Quercetin (0.1 μmol/L in perfusate)	10-min flush at reperfusion onset	Vehicle	1) LVDP; 2) CK; 3) IL-1β; 4) IL-6; 5) TNF-α
18	Kandpal et al. (2025)	Wistar rats (male and female, n = 6/6)	100–150 g	Langendorff perfusion of isolated hearts with 30-min global ischemia and 120-min reperfusion	Thiopental (50 mg/kg, i.p.)	Quercetin (25 and 50 mg/kg), i.p	1 injection 30 min before surgery	Vehicle	1) LVDP; 2) +dP/dtmax; 3)-dP/dtmax; 4) infarct size; 5) LDH; 6) CK-MB; 7) TNF-α; 8) MDA; 9) GSH
19	Bartekova et al. (2010)	Wistar rats (male, n = 6/6)	250–300 g	Langendorff perfusion of isolated hearts with 25-min global ischemia and 120-min reperfusion	Pentobarbital sodium (40 mg/kg i.p.)	Quercetin (15 μmol/L in perfusate)	15 min pre-ischemia and 120 min during reperfusion	Vehicle	1) LVDP; 2) +dP/dtmax; 3)-dP/dtmax; 4) CF; 5) infarct size
20	Gan et al. (2011)	Sprague–Dawley rats (male and female, n = 8/8)	250–300 g	Langendorff perfusion of isolated hearts with 30-min global ischemia and 60-min reperfusion	10% chloral hydrate (1 mL/kg, i.p.)	Quercetin (5 μmol/L in perfusate)	10-min 5 μmol/L quercetin perfusion	Normal saline	1) +dP/dtmax; 2) -dP/dtmax

LVEDP, left ventricular end-diastolic pressure; +dP/dtmax, maximal left ventricular pressure rising rate; -dP/dtmax, maximal left ventricular pressure decreasing rate; TNF, tumor necrosis factor; IL, interleukin; SBP, systolic blood pressure; LVDP, left ventricular developed pressure; CF, coronary flow; RISK, reperfusion injury salvage kinases; FBG, fasting blood glucose; CK, creatine kinase; LDH, lactate dehydrogenase; TUNEL, terminal deoxynucleotidyl transferase dUTP, nick end labeling; p-Akt, phosphorylated protein kinase B; Bcl-2, B-cell lymphoma 2; Bax, Bcl-2-associated X protein; CK-MB, creatine kinase-MB; cTnI, cardiac troponin I; MDA, malondialdehyde; SOD, superoxide dismutase; GSH-Px, Glutathione peroxidase; ROS, reactive oxygen species; AMPK, adenosine monophosphate-activated protein kinase; TSC2, Tuberous sclerosis complex 2; mTOR, mammalian target of rapamycin; i. v., intravenous; i. p., intraperitoneal; i. g., intragastric; p. o., per os.

### 3.3 Risk of bias

Overall, 54.00% of the criteria were marked as “unclear” because basic information on the methodology was missing. All included studies were uniformly rated as ‘unclear' across four methodological criteria: allocation concealment (selection bias), blinding of investigators (performance bias), random outcome assessment (measurement bias), and blinding of outcome assessors (measurement bias). Among these studies, 45.00% reported that animals were randomly grouped and housed in rooms with the same controlled temperature and moderation. One study had a high risk of bias in selective reporting because the results were not fully reported. All studies reported complete outcome data and were considered low-risk for attrition bias; however, two studies had a high risk of bias in baseline characteristics, and one study had a high risk of bias in sequence generation. Because no included study was judged to be at overall low risk of bias, a low-risk-only sensitivity analysis could not be performed. It should be emphasized that an “unclear” risk-of-bias judgment indicates insufficient reporting rather than confirmed methodological failure. However, unclear reporting prevents confident classification of a study as low risk and therefore reduces certainty in the pooled estimates. Although compliance with the Animal Research: Reporting of *In Vivo* Experiments (ARRIVE) guidelines was not scored item by item, the SYRCLE assessment showed incomplete reporting of ARRIVE-relevant items, particularly randomization, allocation concealment, and blinding-related procedures. Accordingly, risk-of-bias-informed sensitivity analyses were restricted to the exclusion of studies with explicit high-risk judgments where outcome-specific data allowed. The results of the quality assessment of the included studies are shown in Table 2.

**TABLE 2:** Risk-of-bias summary of the included studies.

Study	A	B	C	D	E	F	G	H	I	J
Jin et al. (2012)	​	​	​	​	​	​	​	​	​	​
Ferenczyova et al. (2020)	​	​	​	​	​	​	​	​	​	​
Wang Y. et al. (2013)	​	​	​	​	​	​	​	​	​	​
Domingos et al. (2025)	​	​	​	​	​	​	​	​	​	​
Zhang and Qian (2015)	​	​	​	​	​	​	​	​	​	​
Wang X. et al. (2013)	​	​	​	​	​	​	​	​	​	​
Cao et al. (2004)	​	​	​	​	​	​	​	​	​	​
Xuan et al. (2022)	​	​	​	​	​	​	​	​	​	​
Liu et al. (2020)	​	​	​	​	​	​	​	​	​	​
Tao et al. (2023)	​	​	​	​	​	​	​	​	​	​
He et al. (2021)	​	​	​	​	​	​	​	​	​	​
Ahmed et al. (2009)	​	​	​	​	​	​	​	​	​	​
Challa et al. (2011)	​	​	​	​	​	​	​	​	​	​
Brookes et al. (2002)	​	​	​	​	​	​	​	​	​	​
Ikizler et al. (2007)	​	​	​	​	​	​	​	​	​	​
Bartekova et al. (2016)	​	​	​	​	​	​	​	​	​	​
Liu et al. (2021)	​	​	​	​	​	​	​	​	​	​
Kandpal et al. (2025)	​	​	​	​	​	​	​	​	​	​
Bartekova et al. (2010)	​	​	​	​	​	​	​	​	​	​
Gan et al. (2011)	​	​	​	​	​	​	​	​	​	​

A, Sequence generation (selection bias); B, Baseline characteristics (selection bias); C, Allocation concealment (selection bias); D, Random housing (performance bias); E, Blinding investigators (performance bias); F, Random outcome assessment (measurement bias); G, Blinding outcome assessor (measurement bias); H, Incomplete outcome data (attrition bias); I, Selective outcome reporting (reporting bias); J, other sources of bias. Green indicates low risk of bias, yellow indicates unclear risk of bias, and red indicates high risk of bias.

### 3.4 Main results of the meta-analysis

#### 3.4.1 Myocardial infarct size

**FIGURE 2:**
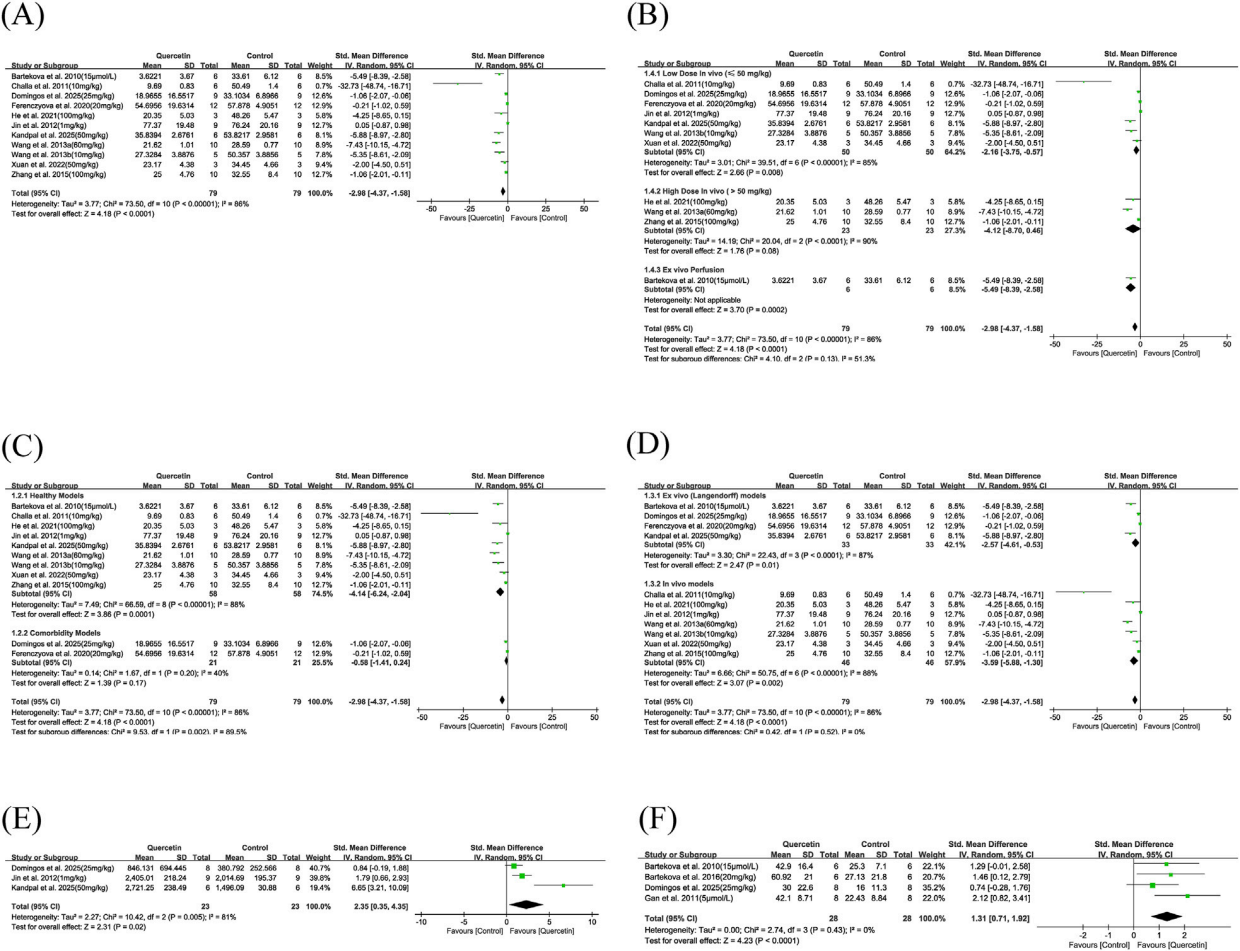
Forest plots of myocardial infarct size and −dP/dtmax outcomes. Note: Forest plots detailing the effects of quercetin on myocardial infarct size and −dP/dtmax-related hemodynamic outcomes. **(A)** Overall forest plot showing the pooled effect size of quercetin on myocardial infarct size reduction; **(B)** Subgroup analysis stratified by dosage regimens; **(C)** Subgroup analysis stratified by the presence of baseline comorbidities; **(D)** Subgroup analysis stratified by experimental model types; **(E)** Absolute −dP/dtmax values; **(F)** −dP/dtmax recovery rate.

Eleven studies involving 158 animals were included in the meta-analysis of infarct size. Quercetin was associated with reduced myocardial infarct size compared with the control group (SMD = −2.98, 95% CI [−4.37, −1.58], P < 0.00001, I^2^ = 86%) (Figure 2A). However, there was considerable heterogeneity across the studies; therefore, sensitivity and subgroup analyses were conducted to explore the potential sources of heterogeneity. Sensitivity analysis using the leave-one-out method showed that excluding any single study yielded no substantial changes in the overall heterogeneity and the pooled effect size. Subsequently, separate subgroup analyses were performed based on dosage (Figure 2B), animal health status (Figure 2C), and model type (Figure 2D). The results showed that only the animal health status subgroup (Healthy vs. Comorbidity models) was statistically significant (P = 0.002). In addition, a substantial reduction in heterogeneity was found in the comorbidity model subgroup (I^2^ = 40%). Subgroup analyses based on dosage (≤50 mg/kg, >50 mg/kg, and *ex vivo* perfusion) and model type (*ex vivo* vs. *in vivo*) did not show statistically significant subgroup differences (P = 0.13 and P = 0.52, respectively), and heterogeneity remained high within most of these subgroups.

To address differences in translational timing, we additionally performed a *post hoc* exploratory subgroup analysis by intervention timing for infarct size. Studies were classified as prevention/preconditioning before I/R, reperfusion-onset/postconditioning treatment, or post-MIRI long-term treatment. Using a random-effects model, the direction of effect favored quercetin in the prevention/preconditioning subgroup (SMD = −2.07, 95% CI [−3.41, −0.73], I^2^ = 86%) and in the reperfusion-onset/postconditioning subgroup (SMD = −8.65, 95% CI [−14.97, −2.33], I^2^ = 82%). The post-MIRI long-term treatment subgroup contained only one study (SMD = −2.00, 95% CI [−4.50, 0.51]). The test for subgroup differences did not show definitive evidence that intervention timing modified the infarct-size effect (P = 0.13). Therefore, this analysis was interpreted descriptively rather than as confirmatory evidence of timing-dependent efficacy. The timing-based subgroup analysis is shown in Supplementary Figure S1.

Additional exploratory study-level analyses were conducted for infarct size. Among systemic-dose studies, log10-transformed quercetin dose was not significantly associated with the infarct-size SMD in univariable random-effects meta-regression (β = −1.20 per 10-fold dose increase, 95% CI −4.61 to 2.20; P = 0.488). The result was similar after excluding Challa et al., 2011 (β = −1.66, 95% CI −4.54 to 1.21; P = 0.256). *Ex vivo* perfusate concentration studies were not included in the continuous dose regression. The analysis is shown in Supplementary Table S1 and Supplementary Figure S2.

Control-group mean infarct size was also not associated with the standardized infarct-size effect (β = 0.56 per 10-percentage-point increase, 95% CI −0.72 to 1.83; P = 0.391), and the finding remained similar after excluding Challa et al., 2011 (β = 0.65, 95% CI −0.47 to 1.76; P = 0.255). Scatter plots likewise showed no clear linear association between control-group infarct size and either the SMD (Pearson r = −0.013, P = 0.969) or the absolute infarct-size reduction (Pearson r = −0.165, P = 0.627). The analysis is shown in Supplementary Table S2 and Supplementary Figure S3.

#### 3.4.2 Myocardial injury biomarkers

**FIGURE 3:**
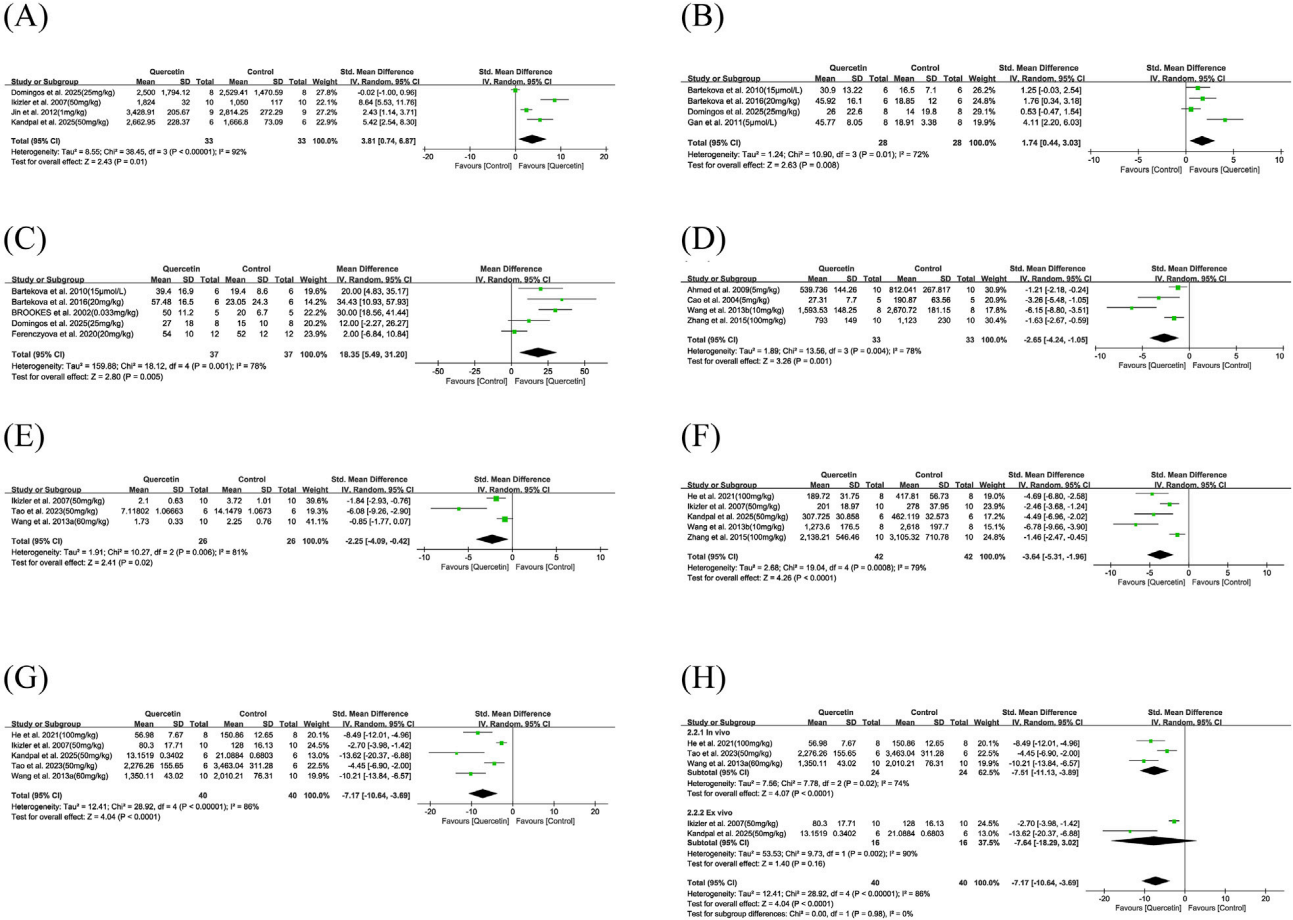
Forest plots of quercetin on + dP/dtmax, LVDP recovery, and myocardial injury biomarkers. Note: The panels display the pooled effect sizes and subgroup analyses for the following indices: **(A)** Absolute +dP/dtmax values; **(B)** +dP/dtmax recovery rate; **(C)** LVDP recovery; **(D)** CK; **(E)** cTnI; **(F)** LDH; **(G)** CK-MB, **(H)** subgroup analysis of CK-MB.

CK, CK-MB, LDH, and cTnI are common markers of myocardial injury and are often measured in combination to monitor the extent of myocardial injury in clinical practice. This meta-analysis indicated that CK (SMD = −2.65, 95% CI [−4.24, −1.05], P = 0.001, I^2^ = 78%, Figure 3D), CK-MB (SMD = −7.17, 95% CI [−10.64, −3.69], P < 0.0001, I^2^ = 86%, Figure 3G), LDH (SMD = −3.64, 95% CI [−5.31, −1.96], P < 0.0001, I^2^ = 79%, Figure 3F), and cTnI (SMD = −2.25, 95% CI [−4.09, −0.42], P = 0.02, I^2^ = 81%, Figure 3E) were reduced after quercetin intervention.

Furthermore, substantial heterogeneity was observed among the studies; therefore, sensitivity and subgroup analyses were performed to explore its potential sources. A leave-one-out sensitivity analysis identified the studies by Wang Y. et al. (2013) and Tao et al. (2023) as the primary sources of heterogeneity for CK and cTnI, respectively. Excluding these studies led to a substantial reduction in heterogeneity (I^2^ decreased to 28% and 46%, respectively). Conversely, for LDH, excluding any single study did not materially change the direction of effect, although heterogeneity remained substantial. Subgroup analysis of CK-MB stratified by experimental model (*in vivo* vs. *ex vivo*) revealed no statistically significant difference (P = 0.98, Figure 3H).

#### 3.4.3 Cardiac hemodynamic function

Hemodynamic indicators, including LVDP, +dP/dtmax, and −dP/dtmax, were used to evaluate short-term post-ischemic cardiac functional recovery in MIRI models. In the revised analysis, cardiac functional outcomes were analyzed according to their reporting metrics. LVDP recovery was pooled as percentage recovery from pre-ischemic baseline in Langendorff-perfused isolated hearts. Compared with the control group, quercetin significantly improved LVDP recovery (MD = 18.35, 95% CI [5.49, 31.20], P = 0.005, I^2^ = 78%, Figure 3C), indicating an 18.35-percentage-point improvement in post-ischemic LVDP recovery rather than an absolute increase in LVDP measured in mmHg. Baseline LVDP values and measurement conditions for studies contributing to this analysis are summarized in Supplementary Table S3. For ±dP/dtmax, absolute values and baseline-adjusted recovery rates were analyzed separately. Quercetin increased absolute + dP/dtmax values (SMD = 3.81, 95% CI [0.74, 6.87], P = 0.01, I^2^ = 92%, Figure 3A) and +dP/dtmax recovery rates (SMD = 1.74, 95% CI [0.44, 3.03], P = 0.008, I^2^ = 72%, Figure 3B). Similarly, quercetin increased absolute −dP/dtmax values (SMD = 2.35, 95% CI [0.35, 4.35], P = 0.02, I^2^ = 81%, Figure 2E) and −dP/dtmax recovery rates (SMD = 1.31, 95% CI [0.71, 1.92], P < 0.0001, I^2^ = 0%, Figure 2F). These results suggest that quercetin was associated with improvements in selected short-term contraction- and relaxation-related indices. However, absolute ±dP/dtmax values and percentage recovery rates were not pooled together because they represent physiologically distinct reporting metrics. Leave-one-out analyses identified Gan et al. (2011) as an influential contributor to heterogeneity in +dP/dtmax recovery rate, with I^2^ decreasing from 72% to 3% after exclusion. For absolute −dP/dtmax, exclusion of Kandpal et al. (2025) reduced I^2^ from 81% to 32%. No clear influential outlier was identified for −dP/dtmax recovery rate, which showed low heterogeneity in the primary analysis.

#### 3.4.4 Oxidative stress

**FIGURE 4:**
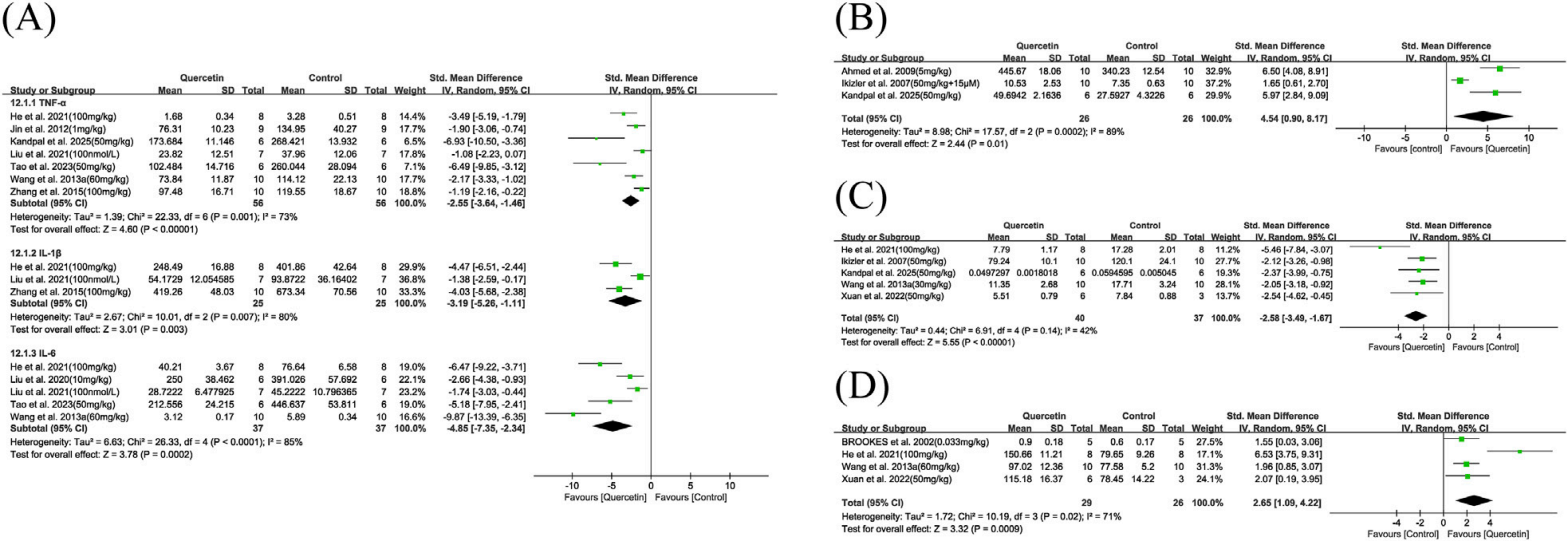
Forest plots of quercetin on inflammatory mediators and oxidative stress indices. Note: The panels display the pooled effect sizes for the following secondary outcomes: **(A)** Composite presentation of pro-inflammatory cytokines (TNF-α, IL-1β, IL-6); **(B)** GSH; **(C)** MDA; **(D)** SOD.

Oxidative stress can be evaluated using oxidative products and antioxidant defenses. MDA levels indicate the degree of lipid peroxidation, whereas SOD and GSH act as critical endogenous antioxidants that scavenge free radicals and prevent oxidative damage. Summarizing the available data on the cardioprotective effects of quercetin, the meta-analysis demonstrated that quercetin intervention significantly reduced MDA levels (SMD = −2.58, 95% CI [−3.49, −1.67], P < 0.0001, I^2^ = 42%, Figure 4C), while notably increasing the levels of SOD (SMD = 2.65, 95% CI [1.09, 4.22], P = 0.0009, I^2^ = 71%, Figure 4D) and GSH (SMD = 4.54, 95% CI [0.90, 8.17], P = 0.01, I^2^ = 89%, Figure 4B) compared to the control group. Given the observed heterogeneity among the studies, a leave-one-out sensitivity analysis was performed to explore its potential sources. The analysis identified the study by Ikizler et al. (2007) as the primary source of variance for GSH; excluding this study completely eliminated the heterogeneity (I^2^ decreased to 0%). Similarly, omitting the study by He et al. (2021) dramatically reduced the heterogeneity to 0% for both MDA and SOD. The present results suggest that quercetin could provide cardioprotection via restoring the oxidative-antioxidant balance.

#### 3.4.5 Pro-inflammatory cytokines

Overexpression of inflammatory markers is a classical hallmark of MIRI, which is intimately associated with myocardial damage in the early stages and subsequent tissue repair. The results of the present meta-analysis demonstrated that quercetin was associated with lower levels of key pro-inflammatory cytokines. Specifically, quercetin was associated with lower levels of TNF-α (SMD = −2.55, 95% CI [-3.64, −1.46], P < 0.001, I^2^ = 73%), IL-1β (SMD = −3.19, 95% CI [-5.26, −1.11], P = 0.003, I^2^ = 80%), and IL-6 (SMD = −4.85, 95% CI [-7.35, −2.34], P < 0.001, I^2^ = 85%) (Figure 4A).

Furthermore, given the presence of substantial inter-study heterogeneity, a leave-one-out sensitivity analysis was conducted to explore potential sources. Notably, for IL-1β, the exclusion of Liu et al. (2021) eliminated the heterogeneity (I^2^ decreased to 0%), suggesting this specific study was the primary contributor to the variance. Conversely, for TNF-α and IL-6, the systematic omission of any single study did not considerably alter the overall heterogeneity or the pooled effect sizes, thereby indicating the statistical robustness of these findings.

### 3.5 Sensitivity analysis

To evaluate the influence of individual studies on the pooled estimates, leave-one-out sensitivity analyses were conducted across all outcome measures. In these analyses, each individual study was sequentially omitted and the pooled effect estimate was recalculated. The direction of effect generally remained unchanged for the assessed outcomes. Detailed leave-one-out results are presented in Supplementary Figure S4.

A split-control sensitivity analysis was additionally performed for myocardial infarct size to evaluate the influence of multi-dose study handling. In this analysis, eligible quercetin monotherapy dose arms from multi-arm studies were retained as separate comparisons, and the sample size of the shared control group was divided across the corresponding comparisons. This analysis included 20 dose-level comparisons and remained significantly in favor of quercetin (SMD = −2.22, 95% CI −3.16 to −1.28, P < 0.00001), with lower but still substantial heterogeneity (I^2^ = 75%) compared with the primary analysis. Compared with the original pooled estimate (SMD = −2.98, 95% CI −4.37 to −1.58; I^2^ = 86%), the effect magnitude was attenuated but the direction of effect remained consistent. The split-control sensitivity analysis is shown in Supplementary Figure S5.

Exploratory risk-of-bias-informed sensitivity analyses were further conducted for outcomes in which exclusion of studies with explicit high-risk judgments still allowed quantitative pooling. First, for total CK, exclusion of Wang Y. et al. (2013) reduced heterogeneity to a low-to-moderate level (I^2^ = 28%), and the pooled effect remained in favor of quercetin (SMD = −1.65, 95% CI −2.49 to −0.81; P = 0.0001) (Supplementary Figure S6A). However, only three contributing studies remained after exclusion; therefore, this result should be interpreted as indicating that Wang Y. et al. (2013) was an influential contributor to heterogeneity rather than as definitive evidence of true homogeneity or high robustness. Second, for LVDP recovery, exclusion of Brookes et al., 2002 yielded a pooled effect that remained favorable to quercetin (MD = 14.40, 95% CI 1.88 to 26.93; P = 0.02) (Supplementary Figure S6B). Nevertheless, residual heterogeneity persisted (I^2^ = 67%), suggesting that LVDP recovery remained influenced by inter-study differences in isolated-heart protocols, baseline function, treatment conditions, and measurement settings.

### 3.6 Publication bias

**FIGURE 5:**
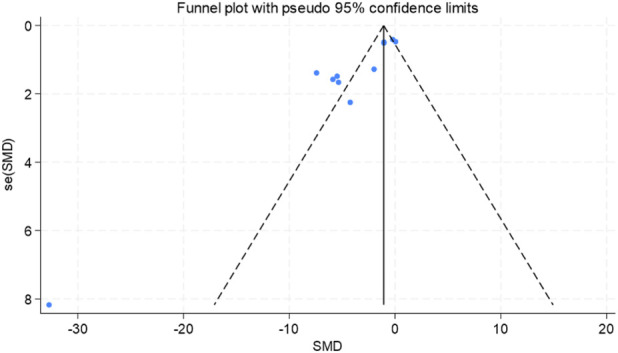
Funnel plot assessing publication bias for myocardial infarct size.

Publication bias can arise when the dissemination of research findings is influenced by the statistical significance or direction of the effect (Hopewell et al., 2009), potentially inflating the estimated efficacy of an intervention. For primary outcomes comprising 10 or more comparisons, publication bias was evaluated using funnel plot analysis and Egger’s regression test for asymmetry. Visual inspection of the funnel plots revealed asymmetry across several outcomes (Figure 5), and Egger’s test confirmed statistically significant asymmetry specifically for the myocardial infarct size endpoint (Table 3). The trim-and-fill analysis was further performed as an exploratory sensitivity analysis. The trim-and-fill procedure did not impute additional studies, and the pooled estimate remained unchanged (Supplementary Table S4; Supplementary Figure S7)**.** Therefore, although trim-and-fill did not alter the summary estimate, the significant Egger test and substantial between-study heterogeneity indicate that the infarct-size effect should still be interpreted cautiously with respect to possible small-study effects or selective reporting.

**TABLE 3:** Egger test.

Outcome	t	p	95% Conf. Interval	
Myocardial infarction size	−6.37	<0.001	−5.601,367	−2.666,504

## 4 Discussion

### 4.1 Summary of evidence

The synthesis of 20 independent studies supports a directionally consistent preclinical cardioprotective signal of quercetin in MIRI models. This signal was reflected by reduced infarct size, lower myocardial injury biomarkers, improvements in selected short-term hemodynamic indices, and modulation of oxidative-stress and inflammatory markers. However, these pooled estimates should be interpreted as evidence of biological plausibility rather than as precise measures of therapeutic efficacy or clinically established benefit.

### 4.2 Mechanistic interpretation and biological plausibility

**FIGURE 6:**
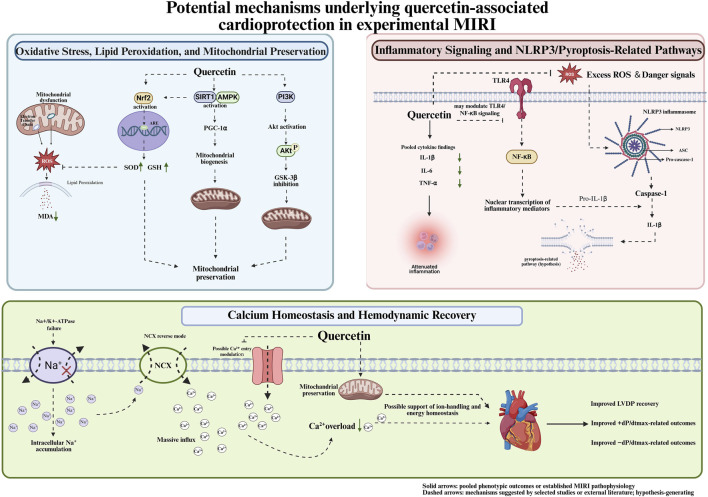
Potential mechanisms underlying quercetin-associated cardioprotection in experimental MIRI.

MIRI is orchestrated by a complex network of pathological processes, including oxidative stress, inflammatory responses, intracellular calcium overload, and regulated cell death pathways (such as apoptosis, pyroptosis, and ferroptosis) (Jin et al., 2024). Given the current lack of satisfactory single-target therapeutic modalities in clinical practice, quercetin has been investigated as a pleiotropic plant-derived flavonoid with potential relevance to several MIRI-related injury pathways. To contextualize the biological plausibility of these findings, we integrated our quantitative meta-analytic results with selected primary-study observations and external mechanistic literature, as illustrated in Figure 6. However, because most included studies measured downstream phenotypic or biomarker outcomes rather than pathway-specific causal endpoints, the mechanistic interpretation below should be regarded as biologically plausible and hypothesis-generating rather than as definitive proof of uniform pathway activation or inhibition across all included studies.

#### 4.2.1 Oxidative stress, lipid peroxidation, and mitochondrial preservation

During the early phase of reperfusion, the explosive burst of ROS from mitochondrial electron transport chain complexes triggers severe subcellular damage (Sagris et al., 2024; Gong et al., 2025). Our meta-analysis provides supportive quantitative evidence that quercetin significantly mitigates lipid peroxidation (evidenced by a reduction in MDA) while preserving endogenous antioxidant reserves (indicated by elevated SOD and GSH). These findings suggest that quercetin may improve redox balance and reduce lipid peroxidation-related injury in experimental MIRI models.

Because excessive lipid peroxidation is also a central feature of ferroptosis (Miyamoto et al., 2022; Zhang et al., 2025), the observed reduction in MDA and preservation of antioxidant capacity may overlap with ferroptosis-associated pathways. However, this review cannot conclude that quercetin directly inhibits ferroptosis, because the included studies did not consistently measure ferroptosis-specific endpoints such as glutathione peroxidase 4 (GPX4) activity, lipid ROS, iron accumulation, acyl-CoA synthetase long-chain family member 4 (ACSL4), or solute carrier family 7 member 11 (SLC7A11). Therefore, ferroptosis-related interpretations should be regarded as mechanistic hypotheses requiring targeted experimental validation.

Beyond direct redox modulation, selected primary studies and external mechanistic literature suggest that quercetin may influence nuclear factor erythroid 2-related factor 2 (Nrf2)/antioxidant response element (ARE) (Zhang et al., 2024; Kandpal et al., 2025) signaling and SIRT1/AMPK-related energy sensing (Liu et al., 2020; Xuan et al., 2022; Dinislam et al., 2025), thereby contributing to mitochondrial preservation. These pathways may be associated with antioxidant enzyme regulation, maintenance of mitochondrial membrane potential, preservation of adenosine triphosphate (ATP) availability, and attenuation of mitochondrial injury in selected experimental models. However, these mechanisms operate on different time scales. Direct free-radical scavenging and mitochondrial ROS modulation are more likely to occur during the early reperfusion phase, whereas Nrf2/ARE-mediated transcriptional responses and peroxisome proliferator-activated receptor gamma coactivator 1-alpha (PGC-1α)-related mitochondrial biogenesis generally require longer exposure (Mata and Cadenas, 2021; Chen et al., 2022). Because the included studies used both acute perfusion/postconditioning protocols and prolonged pretreatment regimens, the present meta-analysis cannot separate immediate chemical antioxidant effects from delayed adaptive genomic responses.

In addition, selected studies have reported involvement of phosphoinositide 3-kinase/protein kinase B (PI3K/Akt)- or reperfusion injury salvage kinase (RISK)-related signaling, including downstream modulation of glycogen synthase kinase-3 beta (GSK-3β)(Wang Y. et al., 2013). These pathways may contribute to mitochondrial preservation and post-ischemic survival signaling. However, PI3K/Akt- or GSK-3β-related findings should not be interpreted as direct evidence of mitochondrial permeability transition pore (mPTP) closure, because the included studies did not systematically assess mPTP opening using mPTP-specific assays such as calcein-cobalt quenching, mitochondrial calcium retention capacity, or standardized mPTP-opening protocols. Thus, mPTP involvement should be considered a plausible downstream hypothesis rather than a definitively established target of quercetin in the present evidence base.

#### 4.2.2 Inflammation, NLRP3 inflammasome, and pyroptosis

The sterile inflammatory response post-ischemia is a central mediator of myocardial damage, with excessive ROS widely recognized as the critical “trigger signal” for the assembly of the NLRP3 inflammasome (comprising NLRP3, apoptosis-associated speck-like protein containing a caspase activation and recruitment domain, and pro-caspase-1) (Coll et al., 2022; Toldo and Abbate, 2024). The assembled inflammasome catalyzes the generation of active caspase-1, which not only processes pro-IL-1β into its mature form but also specifically cleaves the executioner protein Gasdermin D (GSDMD), leading to cardiomyocyte membrane pore formation and ultimate pyroptosis (Shi et al., 2021). Thus, inflammatory priming, inflammasome activation, and pyroptosis-related responses constitute biologically plausible contributors to reperfusion-induced myocardial damage.

In the present meta-analysis, quercetin was associated with reduced levels of pro-inflammatory cytokines, including TNF-α, IL-1β, and IL-6. These findings support an anti-inflammatory signal in experimental MIRI models. At the mechanistic level, selected primary studies suggest that quercetin may modulate ROS-sensitive inflammatory pathways, including Toll-like receptor 4 (TLR4)/nuclear factor-kappa B (NF-κB) signaling and NLRP3-related cytokine processing (He et al., 2021; Tao et al., 2023). This may involve attenuation of NF-κB-dependent inflammatory priming and reduced ROS-dependent inflammasome activation. However, cytokine reduction alone cannot establish direct inhibition of NLRP3 inflammasome assembly, caspase-1 activation, GSDMD-mediated membrane pore formation, or pyroptosis. Therefore, pyroptosis-related interpretations should be considered plausible mechanistic hypotheses rather than conclusions directly demonstrated by pooled cytokine outcomes.

The model context is particularly important for interpreting inflammatory findings. *In vivo* MIRI involves circulating leukocytes, endothelial-cell interactions, neurohumoral regulation, and systemic immune responses. In contrast, *ex vivo* Langendorff heart preparations lack circulating immune-cell recruitment and peripheral inflammatory amplification. Therefore, reductions in IL-1β, IL-6, or TNF-α levels in isolated-heart models should be interpreted as evidence of local myocardial inflammatory signaling rather than systemic immunosuppression or impaired macrophage recruitment.

#### 4.2.3 Calcium homeostasis and hemodynamic recovery

A hallmark of MIRI is intracellular calcium overload, which contributes to systolic dysfunction, diastolic stiffness, and ischemic contracture (Heusch, 2020; Fede et al., 2025). In the present meta-analysis, quercetin was associated with improvements in selected short-term hemodynamic indices, including LVDP recovery and metric-specific +dP/dtmax and −dP/dtmax outcomes. These findings support the possibility that quercetin may help preserve post-ischemic mechanical performance in experimental MIRI models. LVDP recovery reflects developed-pressure recovery normalized to pre-ischemic baseline, whereas +dP/dtmax and −dP/dtmax provide contraction- and relaxation-related pressure-derivative information. These functional improvements are biologically consistent with better energy preservation, reduced calcium overload, and improved maintenance of ion-handling processes reported in selected primary studies (Wang X. et al., 2013; He et al., 2021; Domingos et al., 2025). During MIRI, ischemia-induced energy depletion can impair sarcolemmal Na^+^/K^+^-ATPase activity, promote intracellular Na^+^ accumulation, and favor reverse-mode Na^+^/Ca^2+^ exchanger activity during reperfusion, thereby aggravating Ca^2+^ influx and calcium overload (Wang et al., 2020; Skogestad and Aronsen, 2022). Selected studies suggest that quercetin may help preserve Na^+^/K^+^-ATPase-related ionic homeostasis and mitochondrial energy support for post-ischemic mechanical recovery (He et al., 2021; Domingos et al., 2025). Together, these findings provide a plausible link between the antioxidant/mitochondrial effects of quercetin and improved short-term hemodynamic recovery.

### 4.3 Interpretation of inter-study heterogeneity and statistical robustness

Substantial heterogeneity is expected in preclinical MIRI meta-analyses (Simon-Tillaux et al., 2022; Ineichen et al., 2024) because animal studies differ in strain, sex, age, baseline disease status, ischemia-reperfusion protocol, anesthetic regimen, model setting, route of administration, dose, formulation/vehicle, and intervention timing. Therefore, heterogeneity in this review should be interpreted as reflecting both biological diversity and methodological variation rather than simple statistical inconsistency.

For infarct size, subgroup analyses suggested that baseline animal health status may contribute to heterogeneity. However, this finding should be interpreted cautiously because the comorbidity-relevant evidence was sparse and confounded by strain, age, sex, disease phenotype, ischemia-reperfusion protocol, and intervention timing. The finding should therefore be considered hypothesis-generating rather than definitive evidence that comorbidities attenuate quercetin efficacy.

Infarct size is also susceptible to technical variation. Differences in left anterior descending coronary artery (LAD) ligation site, ischemia and reperfusion duration, temperature control, tissue processing, and 2,3,5-triphenyltetrazolium chloride (TTC)-based planimetry may substantially influence the area at risk and final infarct-size estimates. The infarct-size staining methods, denominators, and image-analysis approaches used in the primary endpoint studies are summarized in Supplementary Table S5. Similarly, *in vivo* and *ex vivo* models differ fundamentally in systemic vascular loading, circulating immune-cell recruitment, afterload, and neurohumoral regulation, making functional recovery indices and cytokine kinetics not directly comparable across model settings (Sutherland and Hearse, 2000; Leivaditis et al., 2024; Yang et al., 2024).

Leave-one-out analyses identified several influential studies contributing to heterogeneity in selected secondary and hemodynamic outcomes. These patterns may reflect study-specific methodological or biological differences rather than contradictory treatment effects. Apparent reductions in I^2^ after removing one study should be interpreted cautiously, especially when only two or three studies remain, because low I^2^ in small evidence pools may reflect limited statistical power rather than true homogeneity. Overall, the direction of effect generally favored quercetin, but this directional consistency should not be equated with precise or highly certain effect estimates.

### 4.4 Limitations

Several limitations should be considered when interpreting the results of this systematic review and meta-analysis.

1. Substantial biological and methodological heterogeneity limits the precision of the pooled estimates. The included studies differed in animal strain, sex, age, disease status, anesthetic regimen, ischemia-reperfusion protocol, model setting, dose, route, formulation/vehicle, and intervention timing. Although subgroup, sensitivity, and exploratory meta-regression analyses were performed where feasible, several strata contained few studies and many factors were confounded. The split-control sensitivity analysis for multi-dose studies showed an attenuated but directionally consistent infarct-size effect, indicating that dose-arm handling may influence effect-size magnitude. Control-group infarct size was not linearly associated with treatment effect in study-level analyses, but this cannot replace animal-level baseline-risk adjustment. Thus, pooled estimates, particularly large SMD values derived from small preclinical studies, should be interpreted as directional summaries rather than protocol-independent or precise treatment-effect estimates.
2. The pharmacological exposure and compound characterization of quercetin remain incompletely defined. Systemic mg/kg dosing and *ex vivo* perfusate concentrations are not directly comparable, and nonlinear, threshold, or U-shaped dose-response patterns, including potential high-concentration pro-oxidant effects, cannot be excluded. Most studies used free quercetin in conventional vehicles or perfusate preparations rather than enhanced delivery formulations, and they generally did not report measurements of parent quercetin, conjugated quercetin metabolites, gut microbiota-derived phenolic metabolites, plasma exposure, or myocardial concentrations. Poor oral bioavailability, first-pass metabolism, rapid conjugation, and interspecies differences in flavonoid metabolism further limit extrapolation to clinically achievable myocardial exposure (Joseph et al., 2022; Mirza et al., 2023). Incomplete reporting of quercetin source, supplier, catalogue or batch number, purity, and analytical confirmation may also reduce reproducibility and pharmacological comparability; therefore, the optimal dose, formulation, therapeutic window, and clinically achievable myocardial exposure cannot be determined.
3. Several outcomes and mechanisms require cautious interpretation. CK, CK-MB, LDH, and cTnI are biologically correlated and should be considered supportive rather than independent confirmatory endpoints. Multiple secondary outcomes were assessed without confirmatory multiplicity-adjusted inference. LVDP recovery, absolute ± dP/dtmax values, and baseline-adjusted recovery rates are physiologically distinct and depend on isolated-heart loading and measurement conditions. Mechanistic interpretations also remain indirect: MDA, SOD, GSH, cytokines, and hemodynamic indices do not establish GPX4-mediated ferroptosis inhibition, direct mPTP blockade, uniform Nrf2/ARE or NLRP3 suppression, sarcoplasmic/endoplasmic reticulum Ca^2+^-ATPase 2a (SERCA2a) restoration, or quercetin-specific mechanisms. In addition, because quercetin is a redox-active plant-derived flavonoid with potential assay-interference or promiscuity-related concerns, particularly in target-based assays (Bolz et al., 2021; Magalhães et al., 2021), the present findings should be interpreted as vehicle-controlled preclinical phenotypic evidence rather than proof of target specificity or clinical efficacy.
4. Certainty and translational validity were limited by study quality and model selection. Allocation concealment, investigator blinding, random outcome assessment, and outcome-assessor blinding were frequently unclear, and TTC-based infarct-size planimetry is operator-dependent. No study was judged to be at overall low risk of bias, and risk-of-bias-informed sensitivity analyses remained exploratory. Funnel-plot asymmetry and significant Egger testing suggested possible small-study effects or selective reporting. Although trim-and-fill analysis did not impute additional studies, this does not exclude publication bias under substantial heterogeneity. *In vivo* experiments also cannot clearly distinguish direct myocardial cytoprotection from indirect systemic vascular actions such as vasodilation, endothelial protection, altered coronary perfusion, or afterload reduction. The evidence was dominated by young, healthy, male or non-sex-stratified rodents, with limited female, aged, comorbid, and no large-animal data; therefore, clinical translation remains uncertain.

### 4.5 Implications for future research and clinical translation

The current evidence suggests that quercetin is biologically active in preclinical MIRI models and may represent a promising cardioprotective candidate. However, future studies should clearly distinguish prophylactic pretreatment from reperfusion-onset therapy, because these strategies correspond to different translational scenarios. Pharmacokinetic studies should report formulation and vehicle details and measure parent quercetin, conjugated quercetin metabolites, plasma exposure, and myocardial exposure under clinically relevant dosing and timing conditions. Future studies should also report quercetin source, supplier, catalogue or batch number, purity, and, where feasible, analytical confirmation of compound identity and quality.

Future experiments should use adequately powered designs including both sexes, clinically relevant age ranges, and common comorbidities such as diabetes, obesity, dyslipidemia, and estrogen deficiency. Studies should follow ARRIVE recommendations, including randomization, allocation concealment, blinded outcome assessment, and standardized infarct-size quantification. Long-term functional and structural endpoints, including ejection fraction, fibrosis, hypertrophy, ventricular remodeling, survival, and progression to heart failure, should be evaluated. Validation in clinically relevant large-animal models should be considered a future research priority.

## 5 Conclusion

This systematic review and meta-analysis indicates that quercetin is associated with a consistent preclinical cardioprotective signal against acute myocardial ischemia-reperfusion injury in rodent *in vivo* and *ex vivo* models. This signal is reflected by reductions in infarct size and myocardial injury biomarkers, improvements in selected short-term functional indices, and modulation of oxidative-stress and inflammatory markers. Quercetin should therefore be regarded as a promising preclinical cardioprotective candidate that requires further pharmacokinetic, mechanistic, and translational validation before clinical application.

## Data Availability

The original contributions presented in the study are included in the article/Supplementary Material, further inquiries can be directed to the corresponding author.
