# Supplementary material for: Quercetin-associated cardioprotection in preclinical myocardial ischemia-reperfusion injury: a systematic review and meta-analysis
Source: Front Pharmacol. 2026 Jul 22;17:1863814. doi: 10.3389/fphar.2026.1863814 (PMC13437634; doi:10.3389/fphar.2026.1863814)
Supplement: Supplementary file 1 [file Supplementaryfile1.docx]

Supplementary Material

# 1 Supplementary Tables

## 1.1  Search strategy for each database

### Search strategy in PubMed

| Steps | Search terms | Results |
| --- | --- | --- |
| #1 | "Quercetin"[Mesh] | 14,532 |
| #2 | (((((((Quercetin[Title/Abstract]) OR (Sophoretin[Title/Abstract])) OR (Meletin[Title/Abstract])) OR (Quercetol[Title/Abstract])) OR (Quercitin[Title/Abstract])) OR (Quertine[Title/Abstract])) OR (3,3',4',5,7-Pentahydroxyflavone[Title/Abstract])) OR (Dikvertin[Title/Abstract]) | 31,450 |
| #3 | Search #1 OR #2 | 33,765 |
| #4 | "Myocardial Reperfusion Injury"[Mesh] | 18,139 |
| #5 | (((((((((((((((((((((((((((((((((Myocardial Reperfusion Injury[Title/Abstract]) OR (Myocardial Reperfusion Injuries[Title/Abstract])) OR (Injury, Myocardial Reperfusion[Title/Abstract])) OR (Injuries, Myocardial Reperfusion[Title/Abstract])) OR (Reperfusion Injury, Myocardial[Title/Abstract])) OR (Reperfusion Injuries, Myocardial[Title/Abstract])) OR (Myocardial Ischemic Reperfusion Injury[Title/Abstract])) OR (Myocardial Ischemic Reperfusion Injuries[Title/Abstract])) OR (Myocardial Ischemia Reperfusion Injury[Title/Abstract])) OR (Myocardial Ischemia Reperfusion Injuries[Title/Abstract])) OR (Myocardial Ischemia/Reperfusion Injury[Title/Abstract])) OR (Myocardial Ischemia/Reperfusion Injuries[Title/Abstract])) OR (Myocardial Ischemia-Reperfusion Injury[Title/Abstract])) OR (Myocardial Ischemia-Reperfusion Injuries[Title/Abstract])) OR (MIRI[Title/Abstract])) OR (Myocardial I/R Injury[Title/Abstract])) OR (Myocardial I/R Injuries[Title/Abstract])) OR (Myocardial IR Injury[Title/Abstract])) OR (Myocardial IR Injuries[Title/Abstract])) OR (Myocardial Ischemia Reperfusion[Title/Abstract])) OR (Myocardial Ischemia/Reperfusion[Title/Abstract])) OR (Myocardial Ischemia-Reperfusion[Title/Abstract])) OR (Myocardial I/R[Title/Abstract])) OR (Ischemia-Reperfusion Injury, Myocardial[Title/Abstract])) OR (Ischemia-Reperfusion Injuries, Myocardial[Title/Abstract])) OR (Cardiac Ischemia-Reperfusion Injury[Title/Abstract])) OR (Cardiac Ischemia-Reperfusion Injuries[Title/Abstract])) OR (Myocardial Reperfusion Damage[Title/Abstract])) OR (Myocardial Reperfusion Damages[Title/Abstract])) OR (Myocardial Revascularization[Title/Abstract])) OR (Myocardial Revascularisation[Title/Abstract])) OR (Myocardial Reperfusion[Title/Abstract])) OR (Reperfusion Arrhythmias[Title/Abstract])) OR (Myocardial Stunning[Title/Abstract]) | 18,502 |
| #6 | Search #4 OR #5 | 29,664 |
| #7 | Search #3 AND #6 | 73 |

Search Strategy in Web of Science

| Steps | Search terms | Results |
| --- | --- | --- |
| #1 | (((((((TS=(Quercetin)) OR TS=(Sophoretin)) OR TS=(Meletin)) OR TS=(Quercetol)) OR TS=(Quercitin)) OR TS=(Quertine)) OR TS=(3,3',4',5,7-Pentahydroxyflavone)) OR TS=(Dikvertin) | 55055 |
| #2 | (((((((((((((((((((((((((((((((((TS=("Myocardial Reperfusion Injury")) OR TS=("Myocardial Reperfusion Injuries")) OR TS=("Injury, Myocardial Reperfusion")) OR TS=("Injuries, Myocardial Reperfusion")) OR TS=("Reperfusion Injury, Myocardial")) OR TS=("Reperfusion Injuries, Myocardial")) OR TS=("Myocardial Ischemic Reperfusion Injury")) OR TS=("Myocardial Ischemic Reperfusion Injuries")) OR TS=("Myocardial Ischemia Reperfusion Injury")) OR TS=("Myocardial Ischemia Reperfusion Injuries")) OR TS=("Myocardial Ischemia/Reperfusion Injury")) OR TS=("Myocardial Ischemia/Reperfusion Injuries")) OR TS=("Myocardial Ischemia-Reperfusion Injury")) OR TS=("Myocardial Ischemia-Reperfusion Injuries")) OR TS=("MIRI")) OR TS=("Myocardial I/R Injury")) OR TS=("Myocardial I/R Injuries")) OR TS=("Myocardial IR Injury")) OR TS=("Myocardial IR Injuries")) OR TS=("Myocardial Ischemia Reperfusion")) OR TS=("Myocardial Ischemia/Reperfusion")) OR TS=("Myocardial Ischemia-Reperfusion")) OR TS=("Myocardial I/R")) OR TS=("Ischemia-Reperfusion Injury, Myocardial")) OR TS=("Ischemia-Reperfusion Injuries, Myocardial")) OR TS=("Cardiac Ischemia-Reperfusion Injury")) OR TS=("Cardiac Ischemia-Reperfusion Injuries")) OR TS=("Myocardial Reperfusion Damage")) OR TS=("Myocardial Reperfusion Damages")) OR TS=("Myocardial Revascularization")) OR TS=("Myocardial Revascularisation")) OR TS=("Myocardial Reperfusion")) OR TS=("Reperfusion Arrhythmias")) OR TS=("Myocardial Stunning") | 50697 |
| #3 | #1 AND #2 | 116 |

Search Strategy in Embase

| Steps | Search terms | Results |
| --- | --- | --- |
| #1 | 'quercetin'/exp OR 'quercetin' | 66584 |
| #2 | 'Quercetin':ab,ti OR 'Sophoretin':ab,ti OR 'Meletin':ab,ti OR 'Quercetol':ab,ti OR 'Quercitin':ab,ti OR 'Quertine':ab,ti OR 'Dikvertin':ab,ti | 39202 |
| #3 | Search #1 OR #2 | 66657 |
| #4 | 'myocardial ischemia reperfusion injury'/exp OR 'myocardial ischemia reperfusion injury' | 12174 |
| #5 | 'myocardial reperfusion injury':ab,ti OR 'myocardial reperfusion injuries':ab,ti OR 'injury, myocardial reperfusion':ab,ti OR 'injuries, myocardial reperfusion':ab,ti OR 'reperfusion injury, myocardial':ab,ti OR 'reperfusion injuries, myocardial':ab,ti OR 'myocardial ischemic reperfusion injury':ab,ti OR 'myocardial ischemic reperfusion injuries':ab,ti OR 'myocardial ischemia reperfusion injury':ab,ti OR 'myocardial ischemia reperfusion injuries':ab,ti OR 'myocardial ischemia/reperfusion injury':ab,ti OR 'myocardial ischemia/reperfusion injuries':ab,ti OR 'myocardial ischemia-reperfusion injury':ab,ti OR 'myocardial ischemia-reperfusion injuries':ab,ti OR 'miri':ab,ti OR 'myocardial i/r injury':ab,ti OR 'myocardial i/r injuries':ab,ti OR 'myocardial ir injury':ab,ti OR 'myocardial ir injuries':ab,ti OR 'myocardial ischemia reperfusion':ab,ti OR 'myocardial ischemia/reperfusion':ab,ti OR 'myocardial ischemia-reperfusion':ab,ti OR 'myocardial i/r':ab,ti OR 'ischemia-reperfusion injury, myocardial':ab,ti OR 'ischemia-reperfusion injuries, myocardial':ab,ti OR 'cardiac ischemia-reperfusion injury':ab,ti OR 'cardiac ischemia-reperfusion injuries':ab,ti OR 'myocardial reperfusion damage':ab,ti OR 'myocardial reperfusion damages':ab,ti OR 'myocardial revascularization':ab,ti OR 'myocardial revascularisation':ab,ti OR 'myocardial reperfusion':ab,ti OR 'reperfusion arrhythmias':ab,ti OR 'myocardial stunning':ab,ti | 24464 |
| #6 | Search #4 OR #5 | 28406 |
| #7 | Search #3 AND #6 | 146 |

Search Strategy in Cochrane Library

| Steps | Search terms | Results |
| --- | --- | --- |
| #1 | MeSH descriptor: [Quercetin] explode all trees | 281 |
| #2 | (Quercetin):ti,ab,kw OR (Sophoretin):ti,ab,kw OR (Meletin):ti,ab,kw OR (Quercetol):ti,ab,kw OR (Quercitin):ti,ab,kw OR (Quertine):ti,ab,kw OR (3,3',4',5,7-Pentahydroxyflavone):ti,ab,kw OR (Dikvertin):ti,ab,kw | 717 |
| #3 | Search #1 OR #2 | 717 |
| #4 | MeSH descriptor: [Myocardial Reperfusion Injury] explode all trees | 634 |
| #5 | (Myocardial Reperfusion Injury):ti,ab,kw OR (Myocardial Reperfusion Injuries):ti,ab,kw OR (Injury, Myocardial Reperfusion):ti,ab,kw OR (Injuries, Myocardial Reperfusion):ti,ab,kw OR (Reperfusion Injury, Myocardial):ti,ab,kw OR (Reperfusion Injuries, Myocardial):ti,ab,kw OR (Myocardial Ischemic Reperfusion Injury):ti,ab,kw OR (Myocardial Ischemic Reperfusion Injuries):ti,ab,kw OR (Myocardial Ischemia Reperfusion Injury):ti,ab,kw OR (Myocardial Ischemia Reperfusion Injuries):ti,ab,kw OR ("Myocardial Ischemia/Reperfusion Injury"):ti,ab,kw OR ("Myocardial Ischemia/Reperfusion Injuries"):ti,ab,kw OR (Myocardial Ischemia-Reperfusion Injury):ti,ab,kw OR (Myocardial Ischemia-Reperfusion Injuries):ti,ab,kw OR (MIRI):ti,ab,kw OR ("Myocardial I/R Injury"):ti,ab,kw OR ("Myocardial I/R Injuries"):ti,ab,kw OR (Myocardial IR Injury):ti,ab,kw OR (Myocardial IR Injuries):ti,ab,kw OR (Myocardial Ischemia Reperfusion):ti,ab,kw OR ("Myocardial Ischemia/Reperfusion"):ti,ab,kw OR (Myocardial Ischemia-Reperfusion):ti,ab,kw OR ("Myocardial I/R"):ti,ab,kw OR (Ischemia-Reperfusion Injury, Myocardial):ti,ab,kw OR (Ischemia-Reperfusion Injuries, Myocardial):ti,ab,kw OR (Cardiac Ischemia-Reperfusion Injury):ti,ab,kw OR (Cardiac Ischemia-Reperfusion Injuries):ti,ab,kw OR (Myocardial Reperfusion Damage):ti,ab,kw OR (Myocardial Reperfusion Damages):ti,ab,kw OR (Myocardial Revascularization):ti,ab,kw OR (Myocardial Revascularisation):ti,ab,kw OR (Myocardial Reperfusion):ti,ab,kw OR (Reperfusion Arrhythmias):ti,ab,kw OR (Myocardial Stunning):ti,ab,kw | 14696 |
| #6 | Search #4 OR #5 | 14696 |
| #7 | Search #3 AND #6 | 0 |

Search Strategy in CNKI

| Steps | Search terms | Results |
| --- | --- | --- |
| #1 | ((SU%=槲皮素 OR SU%=槲皮黄素 OR SU%=栎精) OR (TKA=槲皮素 OR TKA=槲皮黄素 OR TKA=栎精)) AND ((SU%=心肌缺血再灌注损伤 OR SU%=心肌缺血再灌注 OR SU%=缺血再灌注心肌损伤 OR SU%=MIRI OR SU%=心肌I/R损伤 OR SU%=心肌IR损伤 OR SU%=心肌缺血再灌注模型 OR SU%=心肌I/R模型 OR SU%=心肌IR模型) OR (TKA=心肌缺血再灌注损伤 OR TKA=心肌缺血再灌注 OR TKA=缺血再灌注心肌损伤 OR TKA=MIRI OR TKA=心肌I/R损伤 OR TKA=心肌IR损伤 OR TKA=心肌缺血再灌注模型 OR TKA=心肌I/R模型 OR TKA=心肌IR模型)) | 40 |

Search Strategy in WanFang Database

| Steps | Search terms | Results |
| --- | --- | --- |
| #1 | (主题:(槲皮素) or 主题:(槲皮黄素) or 主题:(栎精)) and (主题:(心肌缺血再灌注损伤) or 主题:(心肌缺血再灌注) or 主题:(缺血再灌注心肌损伤) or 主题:(MIRI) or 主题:(心肌I/R损伤) or 主题:(心肌IR损伤) or 主题:(心肌缺血再灌注模型) or 主题:(心肌I/R模型) or 主题:(心肌IR模型)) | 100 |

Search Strategy in VIP Database

| Steps | Search terms | Results |
| --- | --- | --- |
| #1 | ((M=(槲皮素 OR 槲皮黄素 OR 栎精 )) OR (R=(槲皮素 OR 槲皮黄素 OR 栎精))) AND ((M=(心肌缺血再灌注损伤 OR 心肌缺血再灌注 OR 缺血再灌注心肌损伤 OR MIRI OR 心肌I/R损伤 OR 心肌IR损伤 OR 心肌缺血再灌注模型 OR 心肌I/R模型 OR 心肌IR模型)) OR (R=(心肌缺血再灌注损伤 OR 心肌缺血再灌注 OR 缺血再灌注心肌损伤 OR MIRI OR 心肌I/R损伤 OR 心肌IR损伤 OR 心肌缺血再灌注模型 OR 心肌I/R模型 OR 心肌IR模型))) | 45 |

Search Strategy in China Biology Medicine (CBM) Database

| Steps | Search terms | Results |
| --- | --- | --- |
| #1 | "槲皮素"[不加权:扩展] | 4374 |
| #2 | "槲皮素"[常用字段:智能] OR "槲皮黄素"[常用字段:智能] OR "栎精"[常用字段:智能] | 12990 |
| #3 | Search #1 OR #2 | 12990 |
| #4 | "心肌再灌注损伤"[不加权:扩展] | 11367 |
| #5 | "心肌缺血再灌注损伤"[常用字段:智能] OR "心肌缺血再灌注"[常用字段:智能] OR "缺血再灌注心肌损伤"[常用字段:智能] OR "MIRI"[常用字段:智能] OR "心肌I/R损伤"[常用字段:智能] OR "心肌IR损伤"[常用字段:智能] OR "心肌缺血再灌注模型"[常用字段:智能] OR "心肌I/R模型"[常用字段:智能] OR "心肌IR模型"[常用字段:智能] | 14489 |
| #6 | Search #4 OR #5 | 14489 |
| #7 | Search #3 AND #6 | 45 |

## 1.2 Supplementary Table S1

| Analysis | Outcome | β | 95% CI | P |
| --- | --- | --- | --- | --- |
| Meta-regression | SMD | −1.20 | −4.61 to 2.20 | 0.488 |
| Meta-regression excluding Challa 2011 | SMD | −1.66 | −4.54 to 1.21 | 0.256 |

**Supplementary Table S1.** Exploratory dose-response meta-regression for myocardial infarct size.

## 1.3 Supplementary Table S2

| Analysis | Outcome | β | 95% CI | P |
| --- | --- | --- | --- | --- |
| Meta-regression | SMD | 0.56 | −0.72 to 1.83 | 0.391 |
| Meta-regression excluding Challa 2011 | SMD | 0.65 | −0.47 to 1.76 | 0.255 |

**Supplementary Table S2.** Exploratory meta-regression of control-group infarct size and standardized infarct-size effect.

## 1.4 Supplementary Table S3

| Study | Comparison included in LVDP meta-analysis | Baseline LVDP in control group | Baseline LVDP in quercetin group | LVEDP/balloon setting | I/R protocol | LVDP recovery metric |
| --- | --- | --- | --- | --- | --- | --- |
| Bartekova 2010 | Control vs Q1, quercetin before ischemia | 88.2 ± 7.3 mmHg | 85.6 ± 5.9 mmHg | LVEDP adjusted to 3–7 mmHg; hearts with baseline LVDP <60 mmHg excluded | 25 min global ischemia / 120 min reperfusion; functional recovery assessed during first 40 min | % of preischemic baseline |
| Bartekova 2016 | Juvenile control vs juvenile quercetin-treated group | 76.6 ± 7.50 mmHg | 83.72 ± 4.54 mmHg | LVEDP adjusted to 1–5 mmHg | 25 min global ischemia / 40 min reperfusion | % of pre-ischemic baseline |
| Brookes 2002 | Vehicle vs quercetin, 0.033 mg/kg/day | NR | NR | Balloon adjusted to diastolic pressure of 8–10 mmHg; heart paced at 360 beats/min | 22 min global ischemia / 30 min reperfusion; baseline function recorded after 20-min stabilization and final contractile function assessed after 30-min reperfusion | Developed-pressure recovery after I/R |
| Domingos 2025 | OVX vs OVX+Q, 25 mg/kg | NR; baseline inclusion range 80–130 mmHg | NR; baseline inclusion range 80–130 mmHg | LVEDP set to 10 mmHg; hearts with baseline LVDP <80 or >130 mmHg excluded | 30 min no-flow ischemia / 60 min reperfusion | % of pre-ischemic LVDP during stabilization |
| Ferenczyova 2020 | 6-month obese Dia / DiaQ | 83 ± 8 mmHg | 71 ± 3 mmHg | LVEDP adjusted to 1–5 mmHg; constant perfusion pressure 73 mmHg | 30 min global ischemia / 120 min reperfusion; recovery assessed at 40 min | % of pre-ischemic baseline |

**Supplementary Table S3.** Baseline LVDP values and measurement conditions in studies contributing to the LVDP recovery analysis.

Note: Values are presented as originally reported in the primary studies. Only comparisons contributing to the LVDP recovery meta-analysis are shown. For multi-arm studies, only the quercetin arm and its matched control arm used in the quantitative synthesis are listed. Baseline LVDP values were not inferred when not numerically reported. NR, not reported; LVDP, left ventricular developed pressure; LVEDP, left ventricular end-diastolic pressure; OVX, ovariectomized; Dia, diabetic obese ZDF rats; DiaQ, quercetin-treated diabetic obese ZDF rats; I/R, ischemia/reperfusion.

## 1.5 Supplementary Table S4

| Outcome | Model | No. of observed studies | No. of imputed studies | Pooled estimate | 95% Conf. interval |
| --- | --- | --- | --- | --- | --- |
| Myocardial infarction size | Random-effects | 11 | 0 | -2.978 | -4.374 to -1.582 |

**Supplementary Table S4.** Trim-and-fill analysis for myocardial infarct size.

Note: Using the trim-and-fill procedure, no potentially missing studies were imputed for myocardial infarct size. Accordingly, the adjusted pooled estimate was unchanged from the original meta-analysis.

## 1.6 Supplementary Table S5

| Study | Model setting | Staining / quantification method | Infarct-size denominator / normalization | Area at risk reported? | Image-analysis / planimetry / blinding details |
| --- | --- | --- | --- | --- | --- |
| Bartekova 2010 | Ex vivo Langendorff global ischemia-reperfusion | 1% TTC staining; hearts fixed overnight in 10% neutral formaldehyde and cut into 1-mm slices | IS/AR; in global ischemia, AR was defined as the whole LV area | Yes; AR = whole LV | Computerized planimetric method reported; blinding NR |
| Challa 2011 | In vivo regional LAD occlusion/reperfusion; normal and diabetic rats | LV sliced into 2–3 mm sections; 1% TTC in phosphate buffer, 30 min at 37°C; pale necrotic tissue separated and weighed | Percentage of non-viable myocardium of the LV | No formal AAR staining reported | Tissue separation and weighing; image-analysis software NR; blinding NR |
| Jin 2012 | In vivo regional LAD occlusion/reperfusion | LV incubated in 1% TTC in PBS for 15 min at 37°C; infarcted and viable tissues separated | Infarct region / total LV mass | No | Tissue separation and weighing; image-analysis software NR; blinding NR |
| Wang 2013a | In vivo regional LAD occlusion/reperfusion | Evans blue perfusion to identify non-ischemic region; LV cut into 3-mm transverse slices; 1% TTC for 20 min | Infarct area as percentage of AAR mass | Yes; Evans blue used to define AAR | Infarct, AAR, and non-ischemic zones weighed; blinding NR |
| Wang 2013b | In vivo regional ischemia-reperfusion | TTC staining method, based on a referenced infarct-size protocol | Infarct myocardium mass / ischemic risk-zone myocardium mass (%) | Yes; ischemic risk-zone myocardium used as denominator | Weighing-based method implied; image-analysis software NR; blinding NR |
| Zhang and Qian 2015 | In vivo regional LAD occlusion/reperfusion | Heart washed with PBS; ventricles cut into five equal slices below ligation site; 0.1% NBT staining at 37°C for 15 min | Infarct area / total ventricular area (%) | No separate AAR reported | ImagePro Plus 7.0 used; blinding NR |
| Xuan 2022 | In vivo regional ischemia-reperfusion | Myocardial slices 0.1–0.3 cm thick; NBT staining at 37°C for 15 min | Infarcted cross-sectional area / whole LV myocardial cross-sectional area (%) | No separate AAR reported | Image-Pro Plus 4.5 used; blinding NR |
| He 2021 | In vivo regional LAD occlusion/reperfusion | Hearts frozen at −20°C for 30 min, followed by TTC staining; normal myocardium red, infarct region gray-white | Myocardial infarct area percentage | No separate AAR reported | ImageJ used; blinding NR |
| Ferenczyova 2020 | Ex vivo Langendorff global ischemia-reperfusion in ZDF rats | TTC staining after 2 h reperfusion | Infarct size related to AAR; whole LV considered AAR in global ischemia | Yes; AR = whole LV | Image-analysis details NR in available methods/figure caption; blinding NR |
| Domingos 2025 | Ex vivo Langendorff no-flow ischemia/reperfusion in OVX rats | Ventricular portion sliced into 1.5-mm sections; 1% TTC at 37°C for 5 min; fixation in 10% formaldehyde | Percentage of total area of cross-sectional ventricular slices | No separate AAR reported | ImageJ v1.22 used; blinding NR |
| Kandpal 2025 | Ex vivo Langendorff global ischemia-reperfusion | TTC staining; infarcted and viable areas distinguished macroscopically | Infarct-size percentage; denominator not clearly specified in the extractable method text | No separate AAR reported | Macroscopic TTC-based infarct assessment; image-analysis/blinding details NR |

**Supplementary Table S5.** Infarct-size measurement methods in studies contributing to the primary endpoint.

Note: This table summarizes infarct-size measurement methods for studies contributing to the primary endpoint. Among the 20 included studies in the systematic review, 11 provided extractable infarct-size data for quantitative synthesis. Studies not contributing to the infarct-size meta-analysis were not listed in the main body of this table. Abbreviations: AAR/AR, area at risk; IS, infarct size; LAD, left anterior descending coronary artery; LV, left ventricle; NBT, nitro blue tetrazolium; NR, not reported; PBS, phosphate-buffered saline; TTC, 2,3,5-triphenyltetrazolium chloride.

# 2 Supplementary Figures

## 2.1 Supplementary Figure S1

**Supplementary Figure S1.** Post hoc exploratory subgroup analysis of myocardial infarct size by intervention timing.

## 2.2 Supplementary Figure S2

**Supplementary Figure S2.** Exploratory dose-response meta-regression for myocardial infarct size.

Note: The fitted line represents the exploratory univariable random-effects meta-regression.

## 2.3 Supplementary Figure S3

**Supplementary Figure S3.** Exploratory association between control-group infarct size and quercetin treatment effect.

Note: (A) Scatter plot of control-group mean infarct size versus standardized mean difference for infarct size.

(B) Scatter plot of control-group mean infarct size versus absolute infarct-size reduction, calculated as control mean minus quercetin mean. The analysis was restricted to the 11 study-level comparisons included in the primary infarct-size meta-analysis. Control-group mean infarct size was used as a study-level proxy for model injury severity.

## 2.4 Supplementary Figure S4

**Supplementary Figure S4.** Leave-one-out sensitivity analyses for all outcomes.

Note: These panels present the results of the leave-one-out sensitivity analyses for the following outcomes: (A) myocardial infarct size; (B) CK; (C) CK-MB; (D) LDH; (E) cTnI; (F) Absolute +dP/dtmax; (G) +dP/dtmax recovery rate; (H) Absolute −dP/dtmax values; (I) −dP/dtmax recovery rate; (J) LVDP; (K) MDA; (L) SOD; (M) GSH; (N) TNF-α; (O) IL-1β; (P) IL-6.

## 2.5 Supplementary Figure S5

**Supplementary Figure S5.** Split-control sensitivity analysis for myocardial infarct size.

Note: Forest plot showing the split-control sensitivity analysis for myocardial infarct size. For multi-arm studies with multiple eligible quercetin monotherapy dose groups sharing a common control group, each quercetin dose group was retained as a separate comparison, while the shared control-group sample size was divided across the corresponding comparisons. The pooled effect remained significantly in favor of quercetin (SMD = −2.22, 95% CI −3.16 to −1.28), although the effect magnitude was attenuated compared with the primary analysis.

## 2.6 Supplementary Figure S6

**Supplementary Figure S6.** Risk-of-bias-informed sensitivity analyses for selected outcomes.

Note: (A) Forest plot of total CK after exclusion of Wang et al. 2013b, a study with an explicit high-risk judgment in the SYRCLE assessment.

(B) Forest plot of LVDP recovery after exclusion of Brookes et al. 2002, a study with an explicit high-risk judgment in the SYRCLE assessment.

## 2.7 Supplementary Figure S7

**Supplementary Figure S7.** Trim-and-fill funnel plot for myocardial infarct size.

Note: A trim-and-fill analysis was conducted for the myocardial infarct size outcome. No missing studies were imputed, and the adjusted pooled estimate remained unchanged.
